# Draft Genome Sequence of *Streptomyces* sp. XY006, an Endophyte Isolated from Tea (*Camellia sinensis*)

**DOI:** 10.1128/genomeA.00971-17

**Published:** 2017-09-14

**Authors:** Wenna Shan, Huihui Liu, Ying Zhou, Xiaomin Yu

**Affiliations:** a Horticultural Biology and Metabolomics Center, Haixia Institute of Science and Technology, Fujian Agriculture and Forestry University, Fuzhou, China; b College of Horticulture, Fujian Agriculture and Forestry University, Fuzhou, China; c College of Life Sciences, Fujian Agriculture and Forestry University, Fuzhou, China

## Abstract

*Streptomyces* sp. XY006 is an endophytic bacterium isolated from the young leaf material of the tea plant (*Camellia sinensis*). The draft genome consists of 8.2 Mb and encodes 7,415 putative open reading frames. This strain is found to contain a high capacity for the production of natural products.

## GENOME ANNOUNCEMENT

Endophytic bacteria, especially those belonging to the actinomycetes group, are an important yet underdeveloped microbial resource for novel natural products (1–4). As part of a larger research project aimed at discovering new biologically active compounds from endophytic actinomycetes, we isolated *Streptomyces* sp. XY006 from the surface-sterilized young leaf materials of the tea plant (*Camellia sinensis* cv. Tieguanyin). Based on 16S rRNA sequence analysis, strain XY006 showed close similarity to *Streptomyces levis* NRRL B-16370^T^ (99.9% similarity, GenBank accession no. MF496983). The methanol extracts from *Streptomyces* sp. XY006 fermentation were found to be active against several plant fungal pathogens, including *Fusarium graminearum*, *Fusarium oxysporum* f. sp.* lycopersici*, *Fusarium oxysporum* f. sp.* cubense*, and *Colletotrichum* spp. (our unpublished data).

*Streptomyces* sp. XY006 was cultivated on Difco ISP4 agar at 30°C until sporulation. Genomic DNA was extracted using a DNeasy UltraClean microbial kit (Qiagen, USA) following the manufacturer’s protocol. The draft genome was sequenced from an Illumina paired-end library with an average insert size of 350 bp, using an Illumina HiSeq X Ten instrument with a 2 × 150-bp configuration at the Beijing Novogene Bioinformatics Technology Co., Ltd. (Beijing, China). The sequencing of strain XY006 generated 1,891 Mb of data. After adapter filtering and quality trimming, *de novo* assembly of the reads was performed using SOAPdenovo (5) and resulted in 74 scaffolds with an *N*_50_ value of 218,181 bp. The draft genome contains 8,251,847 bp, with a G+C content of 72.33%.

Gene prediction and annotation were performed with the Rapid Annotations using Subsystems Technology (RAST) server (6). A total of 7,415 putative protein-coding genes and 75 RNA genes were identified. Functional annotation of genes revealed that strain XY006 holds potential genes associated with plant growth promotion, including genes involved in indole-3-acetic acid (IAA) synthesis, such as tryptophan 2-monooxygenase and indole-3-acetamide hydrolase (7); the *acdS* gene encoding 1-aminocyclopropane-1-carboxylate deaminase (ACC deaminase); genes involved in mineral phosphate solubilization, transport, and assimilation, such as exopolyphosphatase, inorganic pyrophosphatase, alkaline phosphatase, and some genes in the Pho regulon (8); and genes involved in fungal cell-wall degradation, such as family 19 chitinases (9).

Analysis of the *Streptomyces* sp. XY006 genome with antiSMASH version 4.0 (10) identified 27 putative secondary metabolite biosynthetic gene clusters. Five shared complete identity with previously reported clusters for antimycin, melanin, informatipeptin, albaflavenone, and roseoflavin biosynthesis. The remaining clusters included five nonribosomal peptide synthetases (NRPSs), one type III polyketide synthase (PKS), four PKS-NRPS hybrids, seven terpenes, one NRPS-terpene hybrid, and one bacteriocin.

This work reveals the presence of a wealth of genes related to plant growth promotion and secondary metabolite production in the genome of strain XY006, which merits further studies.

### Accession number(s).

This whole-genome shotgun project has been deposited at DDBJ/ENA/GenBank under the accession number NOKT00000000. The version described in this paper is the first version, NOKT01000000.

## References

[1] Qin S, Xing K, Jiang JH, Xu LH, Li WJ 2011 Biodiversity, bioactive natural products and biotechnological potential of plant-associated endophytic actinobacteria. Appl Microbiol Biotechnol 89:457–473. doi:10.1007/s00253-010-2923-6. 20941490

[2] Golinska P, Wypij M, Agarkar G, Rathod D, Dahm H, Rai M 2015 Endophytic actinobacteria of medicinal plants: diversity and bioactivity. Antonie Van Leeuwenhoek 108:267–289. doi:10.1007/s10482-015-0502-7. 26093915 PMC4491368

[3] Matsumoto A, Takahashi Y 2017 Endophytic actinomycetes: promising source of novel bioactive compounds. J Antibiot (Tokyo) 70:514–519. doi:10.1038/ja.2017.20. 28270688

[4] Nalini MS, Prakash HS 2017 Diversity and bioprospecting of actinomycete endophytes from the medicinal plants. Lett Appl Microbiol 64:261–270. doi:10.1111/lam.12718. 28107573

[5] Luo R, Liu B, Xie Y, Li Z, Huang W, Yuan J, He G, Chen Y, Pan Q, Liu Y, Tang J, Wu G, Zhang H, Shi Y, Liu Y, Yu C, Wang B, Lu Y, Han C, Cheung DW, Yiu SM, Peng S, Xiaoqian Z, Liu G, Liao X, Li Y, Yang H, Wang J, Lam TW, Wang J 2012 SOAPdenovo2: an empirically improved memory-efficient short-read de novo assembler. GigaScience 1:18. doi:10.1186/2047-217X-1-18. 23587118 PMC3626529

[6] Aziz RK, Bartels D, Best AA, DeJongh M, Disz T, Edwards RA, Formsma K, Gerdes S, Glass EM, Kubal M, Meyer F, Olsen GJ, Olson R, Osterman AL, Overbeek RA, McNeil LK, Paarmann D, Paczian T, Parrello B, Pusch GD, Reich C, Stevens R, Vassieva O, Vonstein V, Wilke A, Zagnitko O 2008 The RAST server: rapid annotations using subsystems technology. BMC Genomics 9:75. doi:10.1186/1471-2164-9-75. 18261238 PMC2265698

[7] Manulis S, Shafrir H, Epstein E, Lichter A, Barash I 1994 Biosynthesis of indole-3-acetic acid via the indole-3-acetamide pathway in *Streptomyces* spp. Microbiology 140:1045–1050. doi:10.1099/13500872-140-5-1045. 8025670

[8] Bhattacharyya C, Bakshi U, Mallick I, Mukherji S, Bera B, Ghosh A 2017 Genome-guided insights into the plant growth promotion capabilities of the physiologically versatile *Bacillus aryabhattai* strain AB211. Front Microbiol 8:411. doi:10.3389/fmicb.2017.00411. 28377746 PMC5359284

[9] Gherbawy Y, Elhariry H, Altalhi A, El-Deeb B, Khiralla G 2012 Molecular screening of *Streptomyces* isolates for antifungal activity and family 19 chitinase enzymes. J Microbiol 50:459–468. doi:10.1007/s12275-012-2095-4. 22752910

[10] Weber T, Blin K, Duddela S, Krug D, Kim HU, Bruccoleri R, Lee SY, Fischbach MA, Müller R, Wohlleben W, Breitling R, Takano E, Medema MH 2015 antiSMASH 3.0—a comprehensive resource for the genome mining of biosynthetic gene clusters. Nucleic Acids Res 43:W237–W243. doi:10.1093/nar/gkv437. 25948579 PMC4489286

